# Endoscopic submucosal excavation for gastric plexiform fibromyxoma: A case report and systematic review of literature

**DOI:** 10.3389/fonc.2023.1090259

**Published:** 2023-03-24

**Authors:** Ziqin Xia, Zhidai Zhou, Wei Guo, Hongling Wang, Fan Wang, Feng Zhou

**Affiliations:** ^1^ Department of Gastroenterology, Zhongnan Hospital of Wuhan University, Hubei Clinical Center and Key Laboratory for Intestinal and Colorectal Diseases, Wuhan, China; ^2^ Department of Pathology, Zhongnan Hospital of Wuhan University, Wuhan, China

**Keywords:** plexiform fibromyxoma (PF), endoscopic submucosal excavation (ESE), systematic review, mesenchymal tumor, case report

## Abstract

Plexiform fibromyxoma (PF) is a rare mesenchymal tumor of which the pathogenesis and molecular changes are still unclear. Histologically, it is characterized by a cluster of bland spindle or ovoid cells growing in the mucoid or fibromyxoid stroma rich in small blood vessels. At present, surgical resection is the primary treatment for PF.

## Introduction

Gastric plexiform fibromyxoma (PF) is a rare mesenchymal tumor initially reported as a “plexiform angiomyxoid myofibroblastic tumor (PAMT)” by Takahashi et al. in 2007 (1). In 2010, the World Health Organization (WHO) recommended its current name (2). Due to the lack of typical clinical symptoms, endoscopic manifestations, and imaging features, it is difficult to discriminate PF from gastrointestinal stromal tumors and other gastrointestinal mesenchymal tumors. Therefore,PF is often missed in diagnosis or misdiagnosed as gastrointestinal stromal tumor (GIST) by medical professionals. To date, the diagnosis of PF still relies on histological examination of the lesion. This report aims to deepen the understanding of this rare tumor by presenting the first case ofof endoscopic submucosal excavation (ESE)-based diagnosis of PF. In addition, we performed a systematic review of the literature to better understand this pathology to design appropriate treatment and follow-up strategy for patients with PF.

## Case report

**Figure 1:**
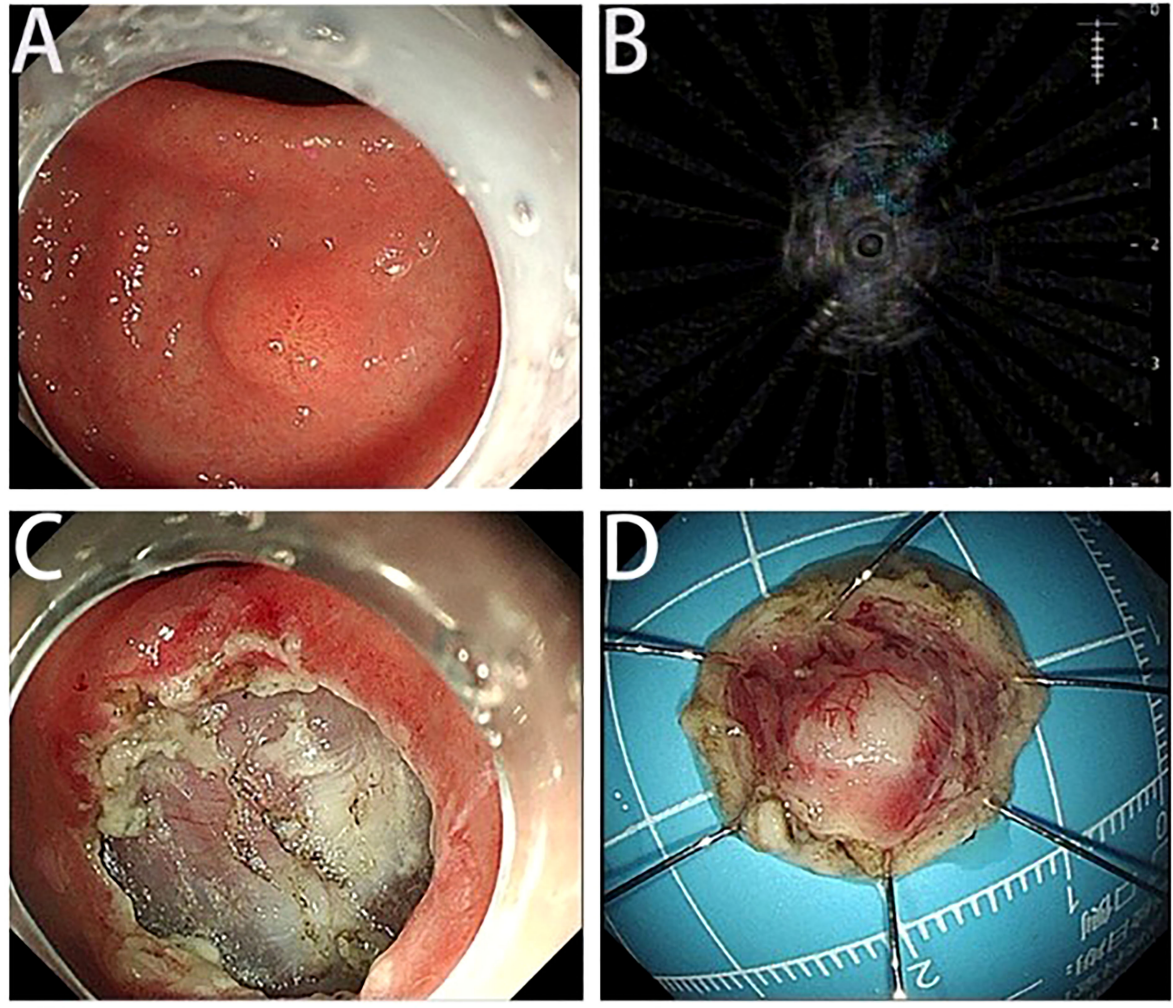
Endoscopic and endoscopic ultrasound findings and ESE images. **(A)** The upper gastrointestinal endoscopy shows a submucosal protrusion at the lesser curvature of gastric antrum. The tumor surface is covered by normal gastric mucosa. **(B)** The EUS shows the hypoechoic lesion originating from the submucosa. **(C)** The wounds after ESE. **(D)** The resected tumor tissue.

**Figure 2:**
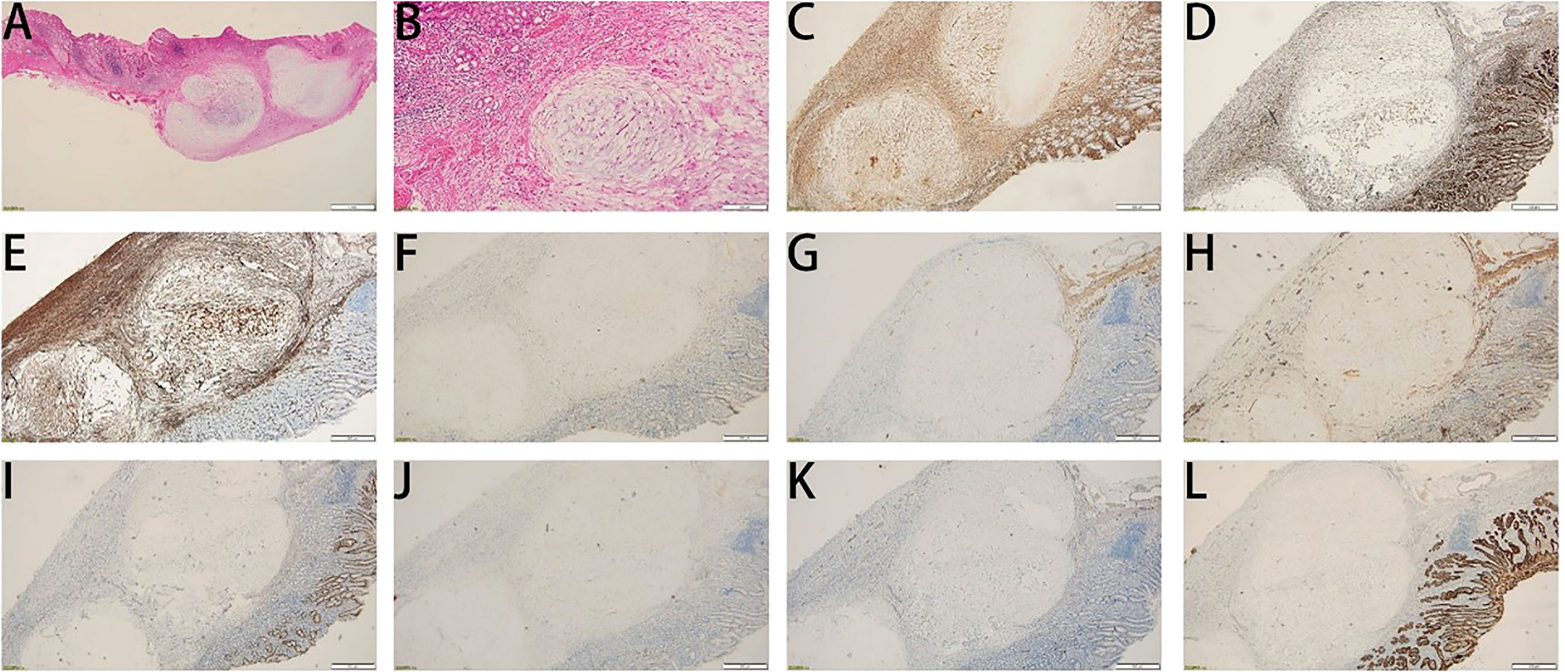
Histological features of the tumor. **(A, B)** Hematoxylin and eosin staining. **(A)** Magnification: 10 ×; **(B)** Magnification: 40 ×. **(C-L)** Immunohistochemical staining for vimentin **(C)**, SDHB **(D)**, CD34 **(E)**, CD117 **(F)**, DEMSIN **(G)**, SMA **(H)**, DOG1 **(I)**, ALK **(J)**, S100 **(K)**, and CK **(L)**.

A 45-year-old woman who suffered from mild abdominal distension and discomfort for more than 10 years received a gastroscopy in the hospital. The result indicated a submucosal apophysis with a rough surface in the lesser curvature of the gastric antrum. The patient was admitted to the Department of Gastroenterology for further examination and treatment. Her medical history showed a mediastinal cyst, thyroid nodule, right breast nodule (BI-RADS type 3), bilateral mammary hyperplasia, and helicobacter pylori infection. Her family history suggested no obvious risks of gastrointestinal tumors. The routine physical examination results seemed normal. The laboratory testing revealed increases in uric acid (377.4 μ mol/l), low-density lipoprotein (3.51mmol/L), thyrotropin (6.0461ulU/ml), Anti-Tg (51.57IU/ml), and A-TPO (233.76IU/mL). No obvious abnormalities were present in the complete blood count, urinalysis, stool analysis, procalcitonin test, and coagulation tests. An enhanced computed tomography scan showed no remarkable changes in the stomach. An upper gastrointestinal endoscopy revealed a submucosal protrusion with a rough surface on the lesser curvature side of the gastric antrum (**Figure 1A**). The lesion was recorded by a real-time 20-MHz ultrasonic probe. An endoscopic ultrasonography (EUS) indicated the clear gastric wall and a hypoechoic lesion located in the gastric submucosa, with a section size of about 7.7 × 4.1mm (**Figure 1B**). Subsequently, ESE was performed to remove the lesion under gastroscopy (**Figures 1C**, **D**): Multiple submucosal injections were conducted at the periphery of the lesion, and the white tumor mass was found to originate from the submucosal layer. Then it was gradually peeled off and completely removed, followed by closure of the wound with hemoclips. Finally the resected lesion size was 1.6*1.5*0.2cm. The pathological examination revealed that the resected tissue was irregular nodular hyperplasia consisting of spindle fibroblasts and myofibroblast-like cells (**Figures 2A, B**). The Immunohistochemistry indicated that the tumor was positive for vimentin, CD34, and SDHB but negative for smooth muscle actin (SMA), Desmin, DOG-1, CD117, cytokeratin, ALK-1, and S-100 protein (**Figures 2C–L**). Approximately 2% of the tumor cells expressed the proliferation marker Ki-67. Based on these findings, the lesion was diagnosed as gastric PF. The patient was alive without any recurrence or metastasis of the tumor after 3 months of follow-up.

## Discussion

**Figure 3:**
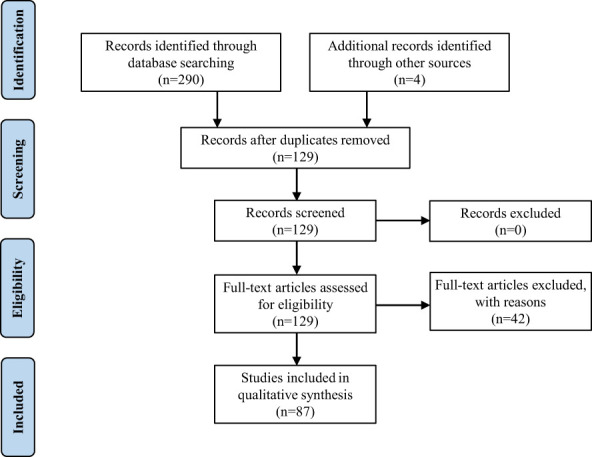
PRISMA flow diagram.

The present study reports the diagnosis of PF for a 45-year-old woman. Due to the rarity and importance of PF, we performed a retrospective review of the literature on which to base the treatment and follow-up of this pathology. Following the Preferred Reporting Items for Systematic Reviews and Meta-Analyses (i.e., “PRISMA”) guidelines, we searched the keywords “plexiform fibromyxoma” and “plexiform angiomyxoid myofibroblastic tumor” in PubMed, Web of Science, Embase, and Cochrane in January 2023, and eventually found 260 articles. Only articles published in English with available abstracts were included. Moreover, we screened the reference lists of relevant studies and identified other 4 potentially eligible studies. Finally, we selected 87 articles including 139 patients (**Supplement Table 1**) as shown in the PRISMA flow diagram (**Figure 3**) (1, 3–88). We extracted the following factors from each article: the first author, date of publication, country, patient number, age, symptoms, tumor size, tumor location, diagnosis, treatment, and follow-up. Data management and analysis were performed using SPSS 26.0 (SPSS Inc., Chicago, IL, USA). The data were presented as proportions for categorical variables, mean ± standard deviation, or median (interquartile range) for continuous variables. The Student’s *t*-test or nonparametric Mann-Whitney U test, where applicable, was used to compare the differences of continuous variables between groups. The Chi-square (χ2) test was used to compare categorical data.

Given the fact that only 33.3% of PF cases were reported by 2015 (approximately 8 years after the initial report of the disorder), the remarkably increased PF incidence in recent years might be attributable to the growing understanding of PF by clinicians and pathologists. The patients’ages ranged from 5 to 81 years (mean age 43.38± 18.26 years; median age 45 years). This age distribution was in approximate accordance with previous reviews (89). Furthermore, PF appeared more frequently in patients between 40-60 years old (38.4%) and 20-40 years old (26.4%). It was rare in patients >75 years old (0.7%). The tumor diameter ranged from 0.8 to 18cm (average 5.23± 3.72 cm), and 61.2% of tumors were 2-6 cm in diameter. Notably, 56.1% of patients were women and 43.9% were men, suggesting a slight female predominance. Interestingly, the age and size distribution of the tumor were significantly independent of gender, as shown by an independent Student’s t-test (*p*=0.774) and Pearson’s chi-squared test(p=0.360). The condition of the tumor surface was reported in 87 cases: 61 (70.1%) were ulcerated, 26(29.9%) were nonulcerated (normal or eroded mucosa). Ulceration of the tumor was significantly associated with hemorrhage-related signs or symptoms (*p*=0.001). The difference in tumor size between ulcerative lesions and nonulcerative lesions was not statistically significant (*p*=0.597).

In 138 reported cases, the most frequent location of PF was the gastric antrum (including pylorus and gastric angle, N=101;73.2%), followed by gastric body (N=16;11.6%), stomach (inside location unspecified, N=6; 4.3%), gastric fundus (N=4;2.9%), duodenum (N=3;2.2%), small bowel(N=3;2.2%), esophagus(N=2; 1.4%), gallbladder (N=1; 0.7%), and mediastinum (N=1; 0.7%).The clinical manifestations ranged from asymptomatic to nonspecific gastrointestinal (GI) symptoms and hemorrhagic gastrointestinal presentations. The most common symptom was abdominal pain. Other clinical presentations included bloating, abdominal discomfort, bleeding, anemia, melena and weight loss. Different manifestations may arise when PF occurred with other diseases or resided in other sites. For example, a 16-year-old girl with mediastinum PF experienced chest pain, shortness of breath, and finger numbness (21). A 35-year-old woman with PF and polycystic ovary syndrome had a cushingoid appearance and amenorrhea (9).

The final diagnosis of PF still relies on histological and immunohistochemical examination of the lesion. Histologically, the typical characteristics of PF include spindle-shaped bland tumor cells arranged characteristically in a plexiform or multinodular pattern, separated by myxoid stroma and rich blood vessels, rare cytological atypia, and mitosis. Immunohistochemistry indicates that PF is diffusively positive for vimentin, muscle-specific actin (MSA), and smooth muscle actin (SMA). The tumor cells may be variably positive for desmin, CD10, and caldesmon. Other markers such as CD117, DOG-1, S100, CD34, β-catenin, anaplastic lymphoma kinase (ALK), and cytokeratin were almost negative. However, how to distinguish between PF and benign lesions before or during surgery to avoid overtreatment is still confusing. Moreover, it is difficult to distinguish PF from other SMT with malignant tendencies such as GIST, the most common SMT (61). EUS is useful to identify SMT and it tells the size, origin layer, echo pattern, lesion margin, and phenotype of SMT (90). Importantly, EUS recognizes the tumor location which is crucial for diagnosis. EUS not only identifies extramural compression and intramural lesions but reveals the nature of lesions (90). In a prospective study of EUS performance, the criteria for predicting malignancy included a tumor size of more than 3 cm, an inhomogeneous pattern, irregular outer margins, and lymph nodes larger than 10 mm in diameter (91). EUS suggests that PF predominantly originates from the submucosal or muscularis propria and is hypoechoic with mild heterogeneity. Hyperechoic lesions were scarce in PF cases (39, 79). However, EUS alone is insufficient to make a definitive diagnosis in most cases, especially those with hypoechoic lesions (90, 91). Therefore, EUS-guided fine needle aspiration (EUS-FNA) plus immunohistochemical analysis is needed for diagnosis. The EUS-based diagnostic traits of GIST include 1) Mildly hypoechoic and well-delineated homogeneous lesion in continuity with the fourth hypoechoic sonographic layer (muscularis propria). 2) EUS-FNA cytology shows that most GIST cells are spindle cells with elongated to wavy nuclei (53). 3) Few GIST tumors show plexiform or nodular growth. 4) GIST tumors are positive for DOG-1 and CD117 and have mutations of the *KIT* or *PDGFRA* gene (92), which are not seen in all the 139 cases involved in our study. It is also necessary to distinguish between PF and gastrointestinal leiomyoma. Gastrointestinal leiomyoma involves esophageal and gastric leiomyomas. EUS shows that gastrointestinal leiomyoma is a well-circumscribed hypoechoic homogeneous lesion in the second (muscularis mucosae) or fourth layer (muscularis propria) (90). Microscopically, a leiomyoma comprises irregular fascicular smooth muscle cells with bright eosinophilic cytoplasm and blunt-ended nuclei. Gastrointestinal leiomyoma is positive for SMA and desmin. Furthermore, PF must be discriminated against schwannomas. Schwannomas are tumors of neural origin and are mainly located in the proximal portion of the stomach. On EUS, schwannomas look similar to PF or GISTs. However, schwannomas are typically diffusively and strongly positive for S100 (93). It should be noted that the results of EUS-FNA could be affected by the tumor size, location, number of punctures, tumor nature, patient condition, and experience of pathologists.

Of all the 139 reported cases, 117 cases offered treatment, with most performed for surgical resection (87.2%), only 11.1% for endoscopic treatment, and two cases adopted to biopsy-only without resection. In our case, we initially considered benign submucosal lesions based on contrast-enhanced CT and EUS. So we decided to remove the tumor through ESE rather than surgical resection. Previous studies reported no recurrence or metastasis of PF after resection in the follow-up, suggesting PF as a benign mesenchymal tumor. Recently, PF recurrence and metastasis (liver and bilateral ovary metastasis) were reported one year after ESD and the patient died 4 months after total gastrectomy (81). This is the first case of fatal PF recurrence and distant metastasis. Furthermore, vascular and viscera invasion have also been reported in individual cases (6, 88). Therefore, whether PF is benign requires further investigation. Follow-up data were available for 88 cases, the uneventful or alive duration ranged from 0.75 to 306 months, with a median of 14 months. We found that surgical resection was performed for all patients with bleeding symptoms (including hematemesis, upper gastrointestinal bleeding, melena), but the difference in treatment between with bleeding symptoms and with non-bleeding patients was not statistically significant(p=0.074). When discussing the influencing factors of treatment methods, we found that the difference in tumor location between surgical and nonsurgical treatment was not statistically significant (P =0.184) while the size and surface condition of lesions were (P=0.001, P=0.005). Due to its good prognosis and based on relevant guidelines on SMT, we recommend that if there are no clinically malignant features, such as irregular margins, ulceration, and/or growth during endoscopic follow-up, gastric PF less than 2 cm in diameter could be removed by endoscopy. If PF is larger than 2 cm in diameter and displays high-risk features and SMT increases in size, surgical resection is highly recommended. It is noteworthy that this case is relatively complicated in the presence of the mediastinal cyst. Whether there is a correlation between the two entities needs further evaluation

In conclusion, gastric PF is an extremely rare mesenchymal tumor. Till now, surgical resection is the primary treatment option for this disease. The final diagnosis of PF depends on the pathology and immunohistochemistry after resection. However, it is always necessary to make preoperative pathological diagnosis for all SMT cases. In this text, EUS - FNA samples and subsequent careful morphological evaluation and immunohistochemical staining make it possible to diagnose PF before or during operation. Previous studies reported no recurrence or metastasis of PF after resection in the follow-up, suggesting PF as a benign mesenchymal tumor. However, the recently reported cases of PF recurrence and distant metastasis and multiple organ invasion deserve our more consideration about whether it is a benign disease. In the future, research regarding the pathogenesis and molecular changes of tumor development and effective treatment methods are required for improving the diagnosis and treatment of PF.

## Data availability statement

The original contributions presented in the study are included in the article/**Supplementary Material**. Further inquiries can be directed to the corresponding author.

## Ethics statement

Written informed consent was obtained from the individual(s) for the publication of any potentially identifiable images or data included in this article.

## Author contributions

ZX and ZZ analyzed the clinical data, and prepared the initial manuscript. ZX and ZZ contributed equally to this study. HW and FW performed the operation together. WG performed pathological diagnosis. FZ designed the study, and revised the manuscript critically. All authors contributed to the article and approved the submitted version.

## Supplementary material

Supplementary Table 1 Characteristics of studies included in the review.
